# Supplementary material for: Leishmania RNA virus-1 is similarly detected among metastatic and non-metastatic phenotypes in a prospective cohort of American Tegumentary Leishmaniasis
Source: PLoS Negl Trop Dis. 2022 Jan 28;16(1):e0010162. doi: 10.1371/journal.pntd.0010162 (PMC8827429; doi:10.1371/journal.pntd.0010162)
Supplement: Attachment — Submitted filename: cover letter revised version.docx [file pntd.0010162.s002.docx]

January 4, 2022

Professor Walderez O. Dutra

Deputy Editor

PLOS Neglected tropical diseases

Dear Professor Dutra,

Re: Manuscript submission: “Leishmania RNA virus-1 is similarly detected among metastatic and non-metastatic phenotypes in a prospective cohort of American Tegumentary Leishmaniasis”

Thank you for reviewing this Research manuscript for publication in PLOS Neglected tropical diseases. We appreciate the reviewer’s feedback. Please see below a detailed response to the reviewer’s comments:

Reviewer #2

1. Methods: The objectives of the study are clear presented. The methods applied were adequate, although better protocols for parasite quantification are already available employing endogenous and exogenous controls. Considering that the Leishmania species might be related to the final prognosis of ATL, to use all them together it is maybe not appropriated. So. I suggest to perform the analysis using L. braziliensis only, as the majority of cases were infected by this species.

**Response:** this manuscript aims to highlight the multifactorial nature of neglected tropical diseases. We agree with reviewers that one determinant of ATL outcomes is the infecting Leishmania species (as stated in lines 82-83), but adverse outcomes are also common among non-*L. (V) braziliensis* infections (lines 84-85) and epidemiologic data suggest other factors influencing outcomes as genetics or environmental conditions (lines 374-377, 408-410). Most pre-clinical models showing the metastatic effect of LRV1 were based in *L. (V) guyanensis* models (lines 383-397), consequently, a similar trend is expected in humans. This trend was not found in this manuscript or in previous reports (lines 359-363), supporting our conclusions that Leishmania species either LRV1 are not major factors driving severity of ATL. Restricting the analysis to L. (V) braziliensis fails to explore the effect of Leishmania species and LRV1 in the pathobiology of ATL. From that perspective, we respectfully consider all Leishmania species should be maintained.

2. Results: The results were well presented, but I think that data showing parasite quantification should be presented, at least as supplementary material. Viral load can be an aspect also related to the severity of the disease and unfortunately this was not tested.

**Response:** parasite load was included in two outcomes of interest (severity - table 2 and treatment failure - table 3) but not in the analysis by phenotype. As requested by the reviewer, it has been included in table 1 and values per case are available in the supplementary material. We agree viral load is a very informative feature but unfortunately this measurement was not considered when the study was designed. Consequently, we are unable to include viral load in this manuscript.

3. Conclusions: The conclusions were supported by the results, but conduction other analysis as suggested above could change the discussion and conclusion. There is a paragraph in the discussion that is not relevant for this article (from line 383 to 410).
   For example, there are few information available on other species than L. braziliensis and L. guyanensnsis. There is a possibility of LRV1-L.(Viannia) co-species evolution and maybe LRV from other species have a different role when interacting with the host. Furthermore, viral load was not well explored in clinical samples, and this might have an important role.

**Response:** As referred in observation 1, we consider restringing analysis to *L. (V) braziliensis* does not tackle the multifactorial nature of ATL, consequently our discussion and conclusions are unchanged. In our response to observation 1 we also justify why lines 383 – 410 are relevant to discuss. The first half of this paragraph summarise the main findings of preclinical studies which are not satisfactorily reproduced in clinical studies. In this paragraph we also discussed non-parasite factors (as host genetics) influencing preclinical studies and not considered by these studies which suggested causation despite the lack of correlation in clinical studies.

4. Editorial and Data Presentation Modifications: results on Parasite load was not demonstrated and was presented very superficially. Raw data should be presented.

**Response:** parasite load was included in the first outcome as recommended by the reviewer and it was available for the other two outcomes in the first submission. Raw data is included as supplementary material.

I hope you find our responses satisfactory, and the manuscript is published after fulfilling PLOS NTD requirements.

Sincerely,

Braulio M. Valencia
